# Clinical Utility of Negative Multiparametric Magnetic Resonance Imaging in the Diagnosis of Prostate Cancer and Clinically Significant Prostate Cancer

**DOI:** 10.1016/j.euros.2021.03.008

**Published:** 2021-04-19

**Authors:** Vinayak G. Wagaskar, Micah Levy, Parita Ratnani, Kate Moody, Mariely Garcia, Adriana M. Pedraza, Sneha Parekh, Krunal Pandav, Bhavya Shukla, Sonya Prasad, Stanislaw Sobotka, Kenneth Haines, Sanoj Punnen, Peter Wiklund, Ash Tewari

**Affiliations:** a Department of Urology, Icahn School of Medicine at Mount Sinai Hospital, New York, NY, USA; b Department of Urology, Pontificia Universidad Javeriana, Hospital Universitario San Ignacio, Bogota, Colombia; c Department of Pathology, Icahn School of Medicine at Mount Sinai Hospital, New York, NY, USA; d Department of Urology, University of Miami, Miller School of Medicine, Miami, FL, USA

**Keywords:** Prostate cancer, Predictive nomogram, Multiparametric magnetic resonance imaging

## Abstract

**Background:**

Multiparametric magnetic resonance imaging (MRI) is increasingly used to diagnose prostate cancer (PCa). It is not yet established whether all men with negative MRI (Prostate Imaging-Reporting and Data System version 2 score <3) should undergo prostate biopsy or not.

**Objective:**

To develop and validate a prediction model that uses clinical parameters to reduce unnecessary prostate biopsies by predicting PCa and clinically significant PCa (csPCa) for men with negative MRI findings who are at risk of harboring PCa.

**Design, setting, and participants:**

This was a retrospective analysis of 200 men with negative MRI at risk of PCa who underwent prostate biopsy (2014–2020) with prostate-specific antigen (PSA) >4 ng/ml, 4Kscore of >7%, PSA density ≥0.15 ng/ml/cm^3^, and/or suspicious digital rectal examination. The validation cohort included 182 men from another centre (University of Miami) with negative MRI who underwent systematic prostate biopsy with the same criteria.

**Outcome measurements and statistical analysis:**

csPCa was defined as Gleason grade group ≥2 on biopsy. Multivariable logistic regression analysis was performed using coefficients of logit function for predicting PCa and csPCa. Nomogram validation was performed by calculating the area under receiver operating characteristic curves (AUC) and comparing nomogram-predicted probabilities with actual rates of PCa and csPCa.

**Results and limitations:**

Of 200 men in the development cohort, 18% showed PCa and 8% showed csPCa on biopsy. Of 182 men in the validation cohort, 21% showed PCa and 6% showed csPCa on biopsy. PSA density, 4Kscore, and family history of PCa were significant predictors for PCa and csPCa. The AUC was 0.80 and 0.87 for prediction of PCa and csPCa, respectively. There was agreement between predicted and actual rates of PCa in the validation cohort. Using the prediction model at threshold of 40, 47% of benign biopsies and 15% of indolent PCa cases diagnosed could be avoided, while missing 10% of csPCa cases. The small sample size and number of events are limitations of the study.

**Conclusions:**

Our prediction model can reduce the number of prostate biopsies among men with negative MRI without compromising the detection of csPCa.

**Patient summary:**

We developed a tool for selection of men with negative MRI (magnetic resonance imaging) findings for prostate cancer who should undergo prostate biopsy. This risk prediction tool safely reduces the number of men who need to undergo the procedure.

## Take Home Message

Men with negative magnetic resonance imaging (MRI) findings pose a diagnostic challenge to physicians as they may have prostate cancer in the absence of accepted clinical predictors. Our model for selection of men with negative MRI findings for biopsy safely reduces the number of men who need to undergo a biopsy procedure.

## 1 Introduction

Multiparametric magnetic resonance imaging (mpMRI) has emerged as a promising tool for guiding prostate biopsy decision-making. The introduction of mpMRI-targeted prostate biopsy has increased the detection of clinically significant disease and reduced the number of unnecessary biopsies and the detection of clinically indolent cancers [1], [2], [3], [4]. The most recent European Association of Urology guidelines [5] recommend against biopsy for men with an abnormal prostate-specific antigen (PSA) and negative mpMRI findings, provided the suspected risk of aggressive cancer is low and the patient has discussed the pros and cons of forgoing biopsy with a doctor. The American Urological Association protocol for prostate MRI [6] raises concern regarding the risk of missing clinically significant prostate cancer (csPCa) on negative MRI examinations. Whether men with negative MRI findings can safely avoid unnecessary prostate biopsies remains unclear. Here we describe the development and validation of a novel risk prediction tool for PCa and csPCa among men with negative MRI. This tool will help to identify men who may safely avoid biopsy, reducing the burden of unnecessary biopsies and overtreatment.

## 2 Patients and methods

### 2.1 Study population

This study was approved by the Institutional Review Board (GCO 19-1711) of the Icahn School of Medicine at Mount Sinai (New York, NY, USA) within the Mount Sinai Health System. We retrospectively reviewed our institution’s prostate biopsy database to extract relevant patient records. Between January 2014 and December 2020, 2100 men underwent a 12-core systematic biopsy performed by a single expert surgeon (A.T.) with 20 yr of experience. The biopsies were performed with a spring-loaded biopsy gun and 18-gauge needles. An experienced genitourinary pathologist (K.H.) reviewed the biopsy samples. Indications for biopsy were one or more of the following: prostate-specific antigen (PSA) >4 ng/ml, a 4Kscore (OPKO Diagnostics, Woburn, MA, USA) of >7%, PSA density of ≥0.15 ng/ml/cm^3^, or suspicious digital rectal examination (DRE).

### 2.2 MRI protocol

#### 2.2.1 Patient preparation

All mpMRI scans were performed before the prostate biopsy procedure. A fasting period of 4 h was advised. Fleet enema was recommended before the examination.

#### 2.2.2 Positioning

Diagnostic mpMRI was conducted with the patient in the supine position.

#### 2.2.3 Technique

Prostate evaluations were conducted using 3-T MRI systems equipped with a phased-array coil (development cohort) or a surface coil (validation cohort). The following sequences were obtained: multiplanar high-resolution T2 fast spin echo (FSE);, axial T1 FSE; axial diffusion-weighted imaging; axial T1 in and out of phase; and axial T1 perfusion before and after contrast injection (8 ml of Gadavist [gadobutrol] and 1 mg of glucagon via intramuscular injection). The mpMPI results were evaluated according to Prostate Imaging-Reporting and Data System version 2 (PI-RADS v2) [7] by a radiologist with more than 5 yr of experience in mpMRI prostate imaging (>250 MRI scans per year) for the development cohort, and by two radiologists with more than 5 yr of experience in mpMRI prostate imaging (>200 MRI scans per year) for the validation cohort.

### 2.3 Inclusion and exclusion criteria

Patients were included in the study if they had negative MRI findings, defined as a PI-RADS v2 score of <3. Exclusion criteria were mpMRI results with a PI-RADS v2 score of ≥3 (*n* = 1740), mpMRI carried out after biopsy (*n* = 40), contraindication for 3-T mpMRI, inadequate image quality on mpMRI (*n* = 30), prior hormone therapy or radiation (*n* = 30), or missing information for clinical variables (*n* = 60). In total, 200 men were eligible for inclusion in the analysis. An external cohort of 182 men with negative MRI findings from the University of Miami (Miami, FL, USA) who underwent biopsy for PSA >4 ng/ml and had a 4Kscore of >7%, PSA density of ≥0.15 ng/ml/cm^3^, suspicious DRE, or a combination of these was used for validation.

### 2.4 Outcome definitions and statistical analysis

For our model, the outcome for predicting PCa was defined as Gleason grade group ≥1 on biopsy. Men with this outcome were considered cases and men showing no cancer on biopsy were considered controls. The outcome for predicting csPCa was defined as Gleason grade group ≥2 on biopsy. Men with this outcome were considered cases and men who showed no cancer on biopsy or with Gleason grade group 1 were considered controls. Descriptive statistics for the two groups were collected. Results for continuous variables are reported as the median and interquartile range (IQR) and were compared using the Mann-Whitney *U* test. Results for categorical variables are reported as the frequency and proportion and were compared using a χ^2^ test, as appropriate. The prediction model included the following variables: age, family history of PCa, history of negative prior biopsy, 4Kscore, DRE findings, and PSA density. Because the 4Kscore test incorporates clinical parameters such as age, family history, DRE, and prior biopsy history, we calculated a matrix of correlation coefficients between the 4Kscore and these predictors. We also conducted variance inflation factor analysis (the inflation in the variance for the parameter estimates due to collinearities among predictors) to evaluate the potential presence of substantial multicollinearity between these predictors in our model.

Analysis of correlation coefficients between predictors, as well as the variance inflation index for the predictors, did not indicate the presence of strong collinearity between the 4Kscore and the other predictors in our model. There were no strong correlations (>0.8) between the 4Kscore and other predictors. Multivariable binary logistic regression analysis was performed for the presence of PCa and csPCa in the development cohort. The nomograms predicting PCa and csPCa were built using the coefficients of the logit function.

Nomogram validation was performed in the validation cohort in two stages. First, receiver operating characteristic curves were plotted for the presence of PCa and csPCa using the same variables used to build the nomogram. Second, calibration plots were generated by grouping cases into deciles according to their nomogram-predicted probability and then comparing the mean prediction for the group with the observed proportion of men with PCa or csPCa. Using nomogram-derived probability cutoffs, we calculated the number of biopsies that could be avoided without missing PCa or csPCa in the validation cohort. Statistical analyses were performed using STATA version 12 (StataCorp, College Station, TX, USA) and SAS version 9.4 (SAS Institute, Cary, NC, USA). All tests were two-tailed with a significance level of *p* < 0.05.

## 3 Results

Of the 200 men in the nomogram development cohort, 35 (18%) were diagnosed with PCa and 165 (82%) were not. The median age was 66 yr (IQR 60–70) and 65 yr (IQR 59–69) for PCa and benign biopsies, respectively. The median PSA was 5.3 ng/ml (IQR 3.5–7.6) and 5.3 ng/ml (IQR 3.4–7.7), median PSAD was 0.13 ng/ml/cm^3^ (IQR 0.11–0.15) and 0.08 ng/ml/cm^3^ (IQR 0.06–0.09), and the median 4Kscore was 19 (IQR 13–28) and 9 (IQR 3–18) for men with PCa and men with benign biopsy, respectively. Of the 35 men with PCa, 19 (54%), 10 (28%), two (6%), two (6%), and two (6%) had Gleason grade group 1, 2, 3, 4, and 5, respectively. Of the 182 men in the validation cohort, 144 (79%) did not have PCa on biopsy. Of the remaining 38 men who had PCa in the validation cohort, 27 (71%), five (13%), one (3%), two (5%), and three (8%) had Gleason grade group 1, 2, 3, 4, and 5, respectively (Table 1).

**Table 1:** Comparison of factors between cases and controls in the development and validation cohorts

Factor	Development cohort			Validation cohort		
	Benign Bx	PCa	*p* value	Benign Bx	PCa	*p* value
Patients, *n* (%)	165 (82)	35 (18)		144 (79)	38 (21)	
Median age, yr (IQR)	65 (59–69)	66 (60–70)	0.913	61 (59–69)	60 (58–70)	0.775
Median PSA, ng/ml (IQR)	5.3 (3.4–7.7)	5.3 (3.5–7.6)	0.112	5.4 (3.9–8.6)	5.9 (4.5–8.4)	0.869
Median PSAD, ng/ml/cm^3^ (IQR)	0.08 (0.06–0.09)	0.13 (0.11–0.15)	0.000	0.09 (0.07–0.10)	0.14 (0.11–0.16)	0.024
Median 4Kscore, points (IQR)	9 (3–18)	19 (13–28)	0.000	12 (4, 21)	22 (13, 34)	0.003
Family history of PCa, *n* (%)			0.034			0.917
Negative	135 (82)	23 (66)		124 (86)	33 (87)	
Positive	30 (18)	12 (34)		20 (14)	5 (13)	
Prior negative biopsy, *n* (%)			0.655			0.556
No	101 (61)	20 (57)		103 (72)	29 (76)	
Yes	64 (39)	15 (43)		41 (28)	9 (24)	
DRE, *n* (%)			0.822			0.467
Normal	93 (56)	19 (54)		107 (74)	26 (68)	
Suspicious	72 (44)	16 (46)		37 (26)	12 (32)	
Gleason grade group, *n* (%)						
0	165 (100)	0		144 (100)	0	
1	0	19 (54)		0	27 (71)	
2	0	10 (28)		0	5 (13)	
3	0	2 (6)		0	1 (3)	
4	0	2 (6)		0	2 (5)	
5	0	2 (6)		0	3 (8)	

Bx = biopsy; PCa = prostate cancer; IQR = interquartile range; PSA = prostate-specific antigen; PSAD = PSA density; DRE = digital rectal examination.

### 3.1 Regression analysis

In univariate analysis, family history of PCa, PSA density, and the 4Kscore emerged as significant predictors of PCa. In the multivariable analysis, family history of PCa, PSA density, and the 4Kscore were significantly associated with PCa and csPCa (all *p* < 0.05; Table 1, Table 2).

**Table 2:** Multivariable analysis predicting PCa and csPCa

Variable	Estimate	Standard error	Odds ratio	*p* value
**Presence of PCa**				
Age	−0.012	0.030	0.988	0.692
Family history of PCa	1.236	0.470	3.443	0.009
Prior negative biopsy	0.278	0.438	1.320	0.526
Digital rectal examination	0.215	0.434	1.240	0.621
PSA density	7.230	2.371	563.804	0.002
4Kscore test	0.044	0.013	1.045	0.001
**Presence of csPCa**				
Age	−0.040	0.041	0.961	0.335
Family history of PCa	1.748	0.645	5.743	0.007
Prior negative biopsy	0.826	0.628	2.285	0.188
Digital rectal examination	0.030	0.644	0.970	0.963
PSA density	4.781	2.546	119.26	0.050
4Kscore test	0.066	0.017	1.068	0.000

PCa = prostate cancer; csPCa = clinically significant PCa; PSA = prostate-specific antigen.

### 3.2 Nomogram to estimate the risk of PCa and csPCa

**Fig. 1:**
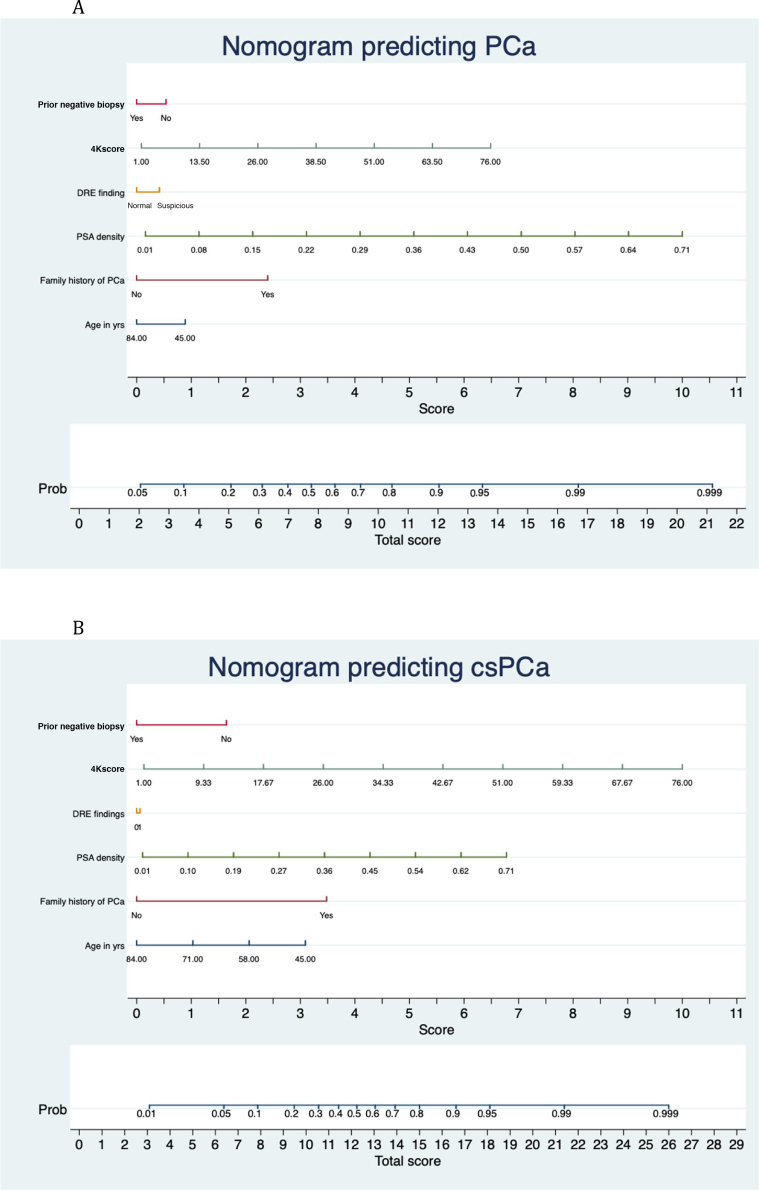
Nomogram for predicting (A) PCa and (B) csPCa at the time of biopsy. Steps for assessing cancer probability from the nomogram are as follows. (1) Locate the patient’s age on the corresponding axis. (2) Draw a line straight down to the score axis to determine how many points towards the probability of cancer the patient is scored for his age. (3) Repeat the process for each additional variable (family history of PCa, DRE, PSA density, 4K score, prior negative biopsy). (4) Calculate the total number of points for the sum of the predictors. (5) Locate the final sum on the total score axis. (6) Draw a line straight up to find the patient’s probability of having cancer. Total scores correspond to a probability value for PCa and csPCa. In B, points 0 and 1 on the scale for DRE finding represent normal and suspicious, respectively. PCa = prostate cancer; csPCa = clinically significant PCa; PSA = prostate-specific antigen; DRE = digital rectal examination; Prob = probability.

Figure 1A shows the nomogram built for prediction of PCa in the development cohort. Family history of PCa, PSA density, and the 4Kscore were significant contributors to the total score determining the probability of PCa in the nomogram.

Figure 1B shows the nomogram built for prediction of csPCa in the development cohort. Family history of PCa, PSA density, and the 4Kscore contributed significantly to the total score.

### 3.3 Nomogram validation

**Fig. 2:**
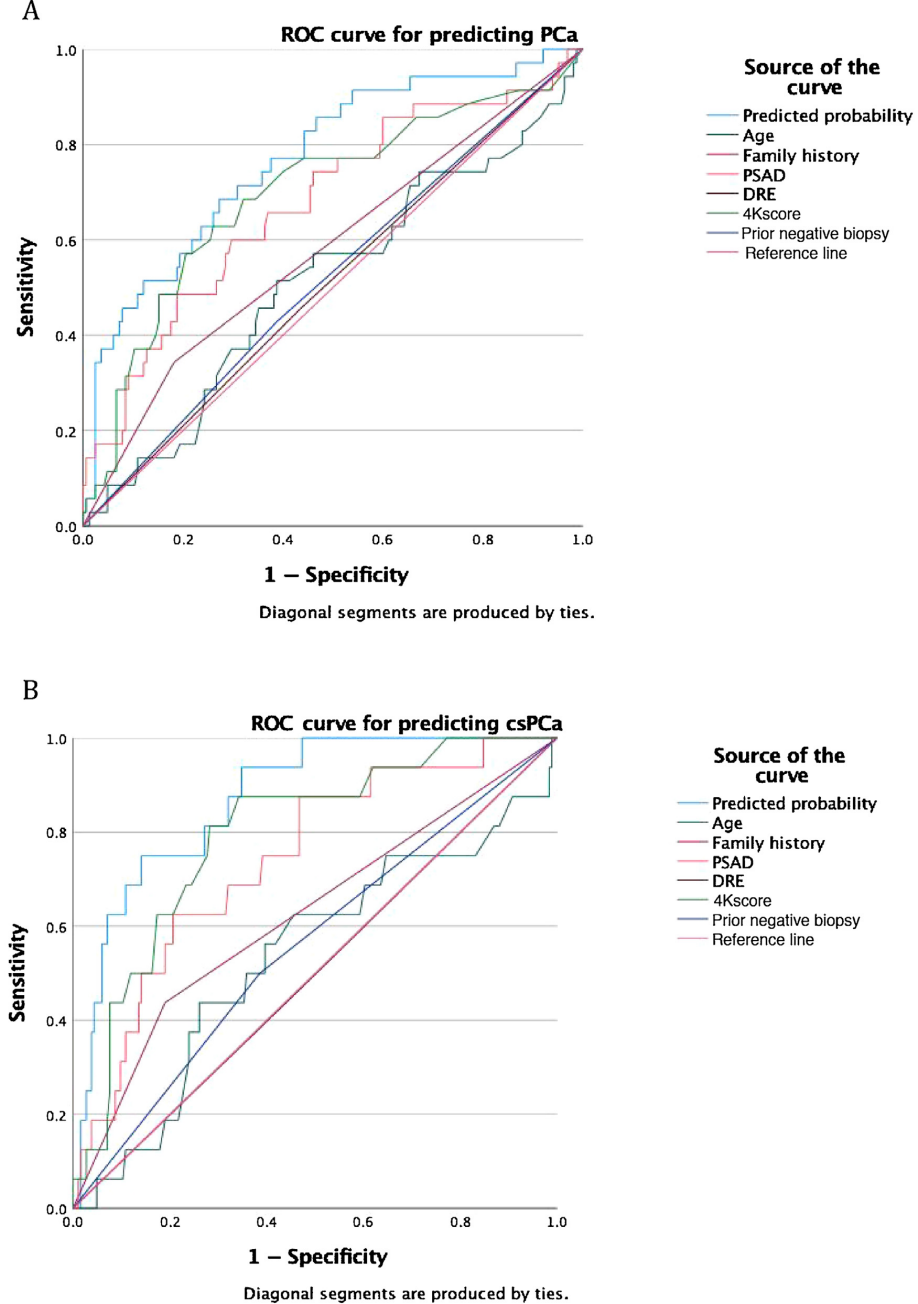
Area under the receiver operating characteristic (ROC) curve for prediction of (A) prostate cancer (PCa) and (B) clinically significant PCa (csPCa) in the validation cohort. PSAD = prostate-specific antigen density; DRE = digital rectal examination

The area under the receiver operating characteristic curve (AUC) for predicting PCa and csPCa was 0.80 and 0.87, respectively (Fig. 2A,B). Here again, the 4Kscore, PSA density, and a family history of PCa were significant contributors to the AUC.

**Fig. 3:**
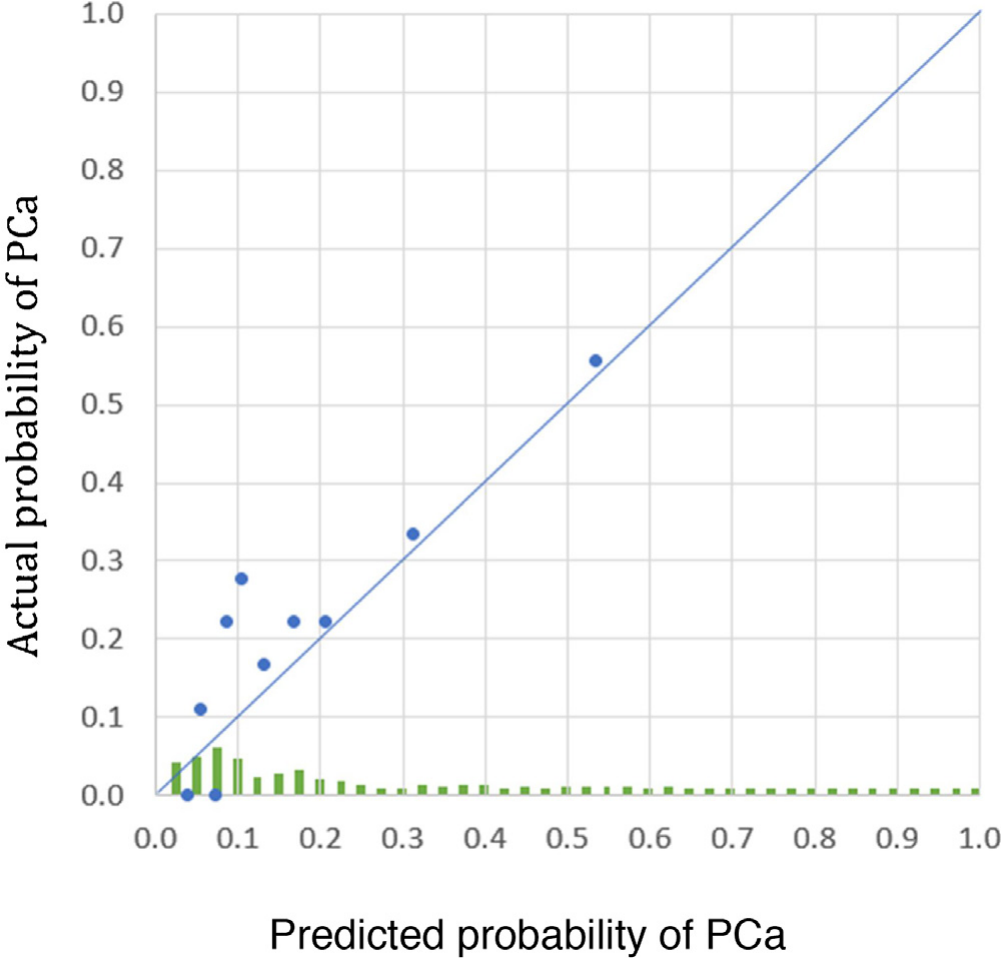
Predicted probability of prostate cancer (PCa) for each case in the validation cohort according to the development model. Each point (average for 18 cases per decile of the nomogram-predicted probability) illustrates the comparison between predicted probability (calculated from the training model) and actual cancer rate for this group of patients in the validation cohort. The diagonal line denotes perfect agreement between the predicted and actual rate of cancer or clinically significant prostate cancer. A histogram of the calculated probability values for the validation cohort is shown along the horizontal axis.

We evaluated the nomogram calibration by comparing predicted and actual probabilities of PCa in the validation cohort. There was agreement between the predicted and actual rate of probabilities for PCa, as seen by points on the diagonal line in Figure 3.

**Fig. 4:**
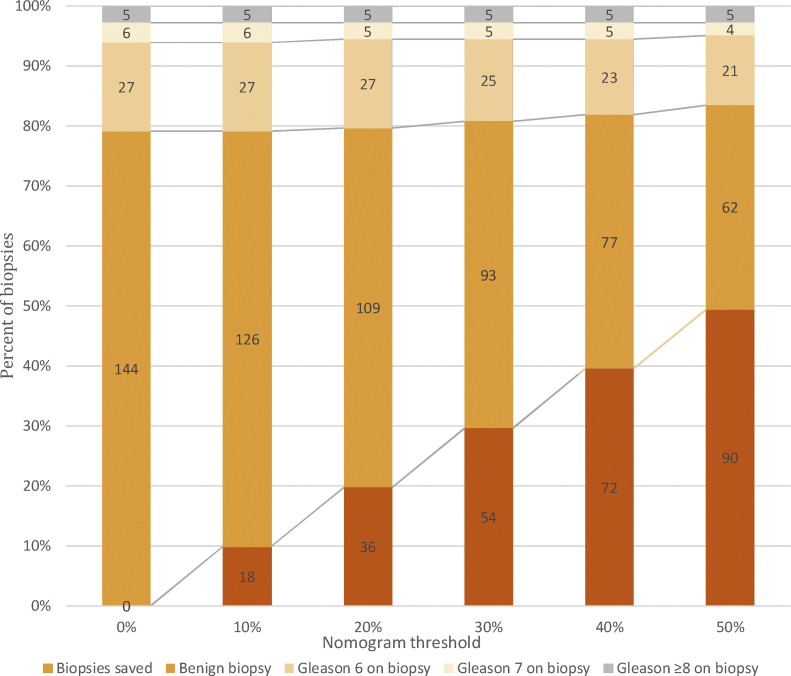
Bar graph showing the number of biopsies that could be avoided in the validation cohort using our model for predicting prostate cancer at various nomogram thresholds.

Using our model in the validation cohort, at a probability threshold of 10%, 10% of overall biopsies could be avoided without missing csPCa, avoiding 14% of benign biopsies (Fig. 4). In addition, at probability thresholds of 20%, 30%, 40%, and 50%, 20%, 30%, 40%, and 50% of overall biopsies could be avoided while missing 10%, 10%, 10%, and 18% of csPCa, avoiding 24%, 35%, 47%, and 57% of benign biopsies, and avoiding diagnosis of 0%, 12%, 15%, and 23% of cases of clinically indolent PCa, respectively.

## 4 Discussion

We developed a multivariable risk prediction tool for patients with negative MRI comprising age, family history of PCa, history of prior negative biopsy, 4Kscore, DRE finding, and PSA density to predict PCa and csPCa. Our model confers three key benefits. (1) The model can be used to avoid a significant number of biopsies among men with negative MRI without compromising detection of csPCa. (2) The model shows the efficacy of the 4Kscore, PSA density, and family history of PCa for predicting PCa and csPCa for men with negative MRI. (3) MRI misses 8% of csPCa cases. As diagnosis of csPCa is critical, the model addresses this gap.

Our model results support the avoidance of a substantial number of biopsies without significantly missing csPCa among men with negative MRI. With the number of prostate biopsy procedures increasing every year, the complications associated with biopsy have attracted greater attention. Common nonfatal complications after biopsy include pain, bleeding, and voiding dysfunction. Postbiopsy fever and infection are less common, but can be potentially fatal complications [8] as there is increasing prevalence of biopsy-related antibiotic-resistant bacterial infections [9]. In addition, standard systematic prostate biopsy is associated with greater detection of indolent or clinically insignificant PCa [10]. Use of our model can help clinicians to reduce unnecessary biopsies among men with negative MRI.

A number of prediction calculators for diagnosing csPCa have been developed. Most of these tools include mpMRI as a variable, yet it remains unclear whether patients with negative MRI should undergo biopsy. Our findings show that men with elevated PSA density, elevated 4Kscore, and a family history of PCa should undergo prostate biopsy regardless of negative MRI findings. Elevated PSA density (≥0.15 ng/ml/cm^3^) in men with negative MRI predicts csPCa [11]. Our previously published study [12] demonstrated the significance of an elevated 4Kscore and PSA density in predicting csPCa in patients with negative MRI. Similar to our study, Buisset et al [13] showed the significance of family history of PCa and PSA density for predicting csPCa in men with negative MRI.

In the literature, the number of csPCa cases missed on negative MRI ranges from 4% to 18% [11], [14]. In our development cohort, 8% of csPCa cases would have been missed without a biopsy based on negative MRI findings, which is within the current expected range for missed csPCa. The long-term prognosis for PCa with deferred treatment is well predicted by the Gleason grade. Egevad et al [15] studied 305 men diagnosed with PCa for whom there was no curative treatment. They found that mean disease-specific survival was 5–10 yr for patients with csPCa (Gleason score ≥7), and 16–20 yr for men with clinically indolent PCa. Moreover, the cribriform pattern on histology seen in csPCa is a strong predictor of distant metastases and disease-specific death [16], with median time to disease-specific death of 120 mo. Clearly, we should make every attempt to diagnose csPCa.

We recognize that our study has some limitations. First, the small sample size and the number of events in the development (16 csPCa cases) and validation (11 csPCa cases) cohorts is a limitation of the study and may affect its applicability. Second, this is a retrospective, single-center study with a single biopsy expert and our outcomes may not be reproducible. In addition, the small number of events and the risk of overfitting for the proposed model with six variables may affect its generalizability and applicability. The *p* values were not corrected for multiple-hypothesis testing. However, our prediction tool was validated in an entirely different cohort to show the robustness of the risk estimation. Finally, we have not revised mpMRI in men with csPCa.

## 5 Conclusions

We developed an easily accessible tool to help clinicians in biopsy decision-making and in counseling patients at risk of PCa with negative MRI. Use of this novel prediction model can significantly reduce the number of biopsies without markedly missing csPCa in men with a negative MRI examination. Our results show the significance of the 4Kscore, PSA density, and family history of PCa for predicting PCa and csPCa in men with negative MRI findings. The prediction model we have developed could provide valuable information for physicians and patients in assessing an individual’s risk for csPCa with negative MRI.

  ***Author contributions***: Vinayak G. Wagaskar had full access to all the data in the study and takes responsibility for the integrity of the data and the accuracy of the data analysis.

  *Study concept and design*: Tewari, Wagaskar.

*Acquisition of data*: Wagaskar, Levy, Moody, Ratnani, Garcia.

*Analysis and interpretation of data*: Wagaskar, Sobotka, Ratnani.

*Drafting of the manuscript*: Wagaskar, Tewari, Wiklund, Levy, Punnen, Moody, Parekh, Pandav, Shukla, Pedraza, Prasad, Haines.

*Critical revision of the manuscript for important intellectual content*: Wagaskar, Wiklund.

*Statistical analysis*: Wagaskar, Ratnani, Sobotka.

*Obtaining funding*: None.

*Administrative, technical, or material support*: None.

*Supervision*: Wagaskar.

*Other*: None.

  ***Financial disclosures:*** Vinayak G. Wagaskar certifies that all conflicts of interest, including specific financial interests and relationships and affiliations relevant to the subject matter or materials discussed in the manuscript (eg, employment/affiliation, grants or funding, consultancies, honoraria, stock ownership or options, expert testimony, royalties, or patents filed, received, or pending), are the following: None.

  ***Funding/Support and role of the sponsor*:** None.

  ***Acknowledgments***: We thank Ms. Sima Rabinowitz for editorial assistance.

## CRediT authorship contribution statement

**Vinayak G. Wagaskar:** Conceptualization, Methodology, Software, Writing - original draft. **Micah Levy:** Data curation, Writing - original draft. **Parita Ratnani:** Methodology, Software. **Kate Moody:** Data curation, Writing - original draft. **Mariely Garcia:** Data curation. **Adriana M. Pedraza:** Writing - original draft. **Sneha Parekh:** Writing - original draft. **Krunal Pandav:** Writing - original draft. **Bhavya Shukla:** Writing - original draft. **Stanislaw Sobotka:** Methodology, Software. **Kenneth Haines:** Data curation. **Sanoj Punnen:** Validation, Writing - review & editing. **Peter Wiklund:** Visualization, Investigation. **Ash Tewari:** Supervision, Investigation, Writing - review & editing.
